# Cancer incidence amongst UK firefighters

**DOI:** 10.1038/s41598-022-24410-3

**Published:** 2023-01-10

**Authors:** Taylor A. M. Wolffe, Andrew Robinson, Kathryn Dickens, Louis Turrell, Anna Clinton, Daniella Maritan-Thomson, Miland Joshi, Anna A. Stec

**Affiliations:** 1 Centre for Fire and Hazards Sciences, University of Central Lancashire, Preston, Lancashire, PR1 2HE UK; 2 Royal Preston Hospital, Lancashire Teaching Hospitals NHS Foundation Trust, Preston, Lancashire, PR2 9HT UK; 3 Lancashire Clinical Trials Unit, University of Central Lancashire, Preston, Lancashire, PR1 2HE UK

**Keywords:** Cancer prevention, Environmental sciences, Risk factors

## Abstract

Firefighters suffer an increased risk of cancer from exposures to chemicals released from fires. Our earlier research has found that fire toxicants not only remain on firefighters’ PPE, but are also tracked back to fire stations. The UK Firefighter Contamination Survey assesses firefighters’ risk of developing cancer due to occupational exposure to fire toxins. Over 4% of surveyed firefighters were found to have a cancer diagnosis, with the age-specific cancer rate up to 323% higher (35–39 year olds) than that of the general population. Firefighters who had served ≥ 15 years were 1.7 times more likely to develop cancer than those who had served less time. Firefighters were at least twice as likely to be diagnosed with cancer if they noticed soot in their nose/throat (odds ratio (OR) = 2.0, 1.1–3.5), or remained in their PPE for more than four hours after attending a fire incident (OR = 2.3, 1.1–5.2). Also associated with an increased likelihood of cancer was: eating while wearing PPE (OR = 1.8, 1.2–2.7); failing to store clean/dirty PPE separately (OR = 1.3, 1.0–1.7); working in a station that smells of fire (OR = 1.3, 1.0–1.8) or not having designated (separated) clean and dirty areas (OR = 1.4, 1.1–1.7); using an on-site washing machine to launder fire hoods (OR = 1.3, 1.0–1.7); feeling that cleaning is not taken seriously at work (OR = 1.5, 1.2–2.0).

## Introduction

Firefighters continue to suffer chronic illnesses as a result of occupational exposure to fire toxins^1–5^. Research has found that carcinogens from fire incidents not only remain on firefighters’ personal protective equipment (PPE), but are also tracked back to fire stations^3,6–8^. These “contaminants” are receiving increasing attention from Fire and Rescue Services globally, and are the focus of decontamination procedures which aim to minimise firefighters’ chronic exposure to toxic fire effluents^9,10^.

To date, the majority of research concerning firefighters’ chronic exposure to contaminants has focused on the context of US firefighters^6,7,11^. Comparatively little research characterises the occupational health of UK firefighters. This research gap has seen the UK’s Industrial Injuries Advisory Council (IIAC) fail to recommend cancer (except mesothelioma) as a prescriptive disease in firefighters^12^. Thus, presumption cannot be assumed for firefighters suffering cancer in the UK, compromising their access to workplace compensation. This is in contrast to some states in the US and Canada, where presumptive legislation protects firefighters’ right to compensation for a range of cancers and other chronic illnesses^13^.

A lack of research concerning UK firefighters’ occupational health also poses challenges for the effective implementation of preventative measures, i.e. measures which would limit chronic exposure to harmful contaminants in the first instance. Without an established baseline, it is difficult to implement or quantify the effectiveness of such preventative measures. Similarly, for UK Fire and Rescue Services (FRSs) required to operate under increasingly strained resources^14^, a lack of current research makes it difficult to prioritise such measures in a resource-efficient manner.

The UK Firefighter Contamination Survey was conducted in order to provide an evidence base for understanding the risks and common sources of contaminant exposure, informing decontamination and future research recommendations. The survey probed UK firefighters’ experiences and behaviours on a range of topics including their health, exposure to fire toxins (duration, frequency etc.), contamination and decontamination practices, PPE (provision, maintenance, cleaning, storage, fit etc.)^8^, attitude/culture, awareness and training^15^.

This manuscript presents links between firefighters’ occupational exposure to fire toxins and their risk of developing cancer.

## Methods

### Survey design

The methods used to conduct the survey and analyse its results are detailed in Wolffe et al., 2023 (i.e. “Contamination of UK Firefighters Personal Protective Equipment and Workplaces”^8^). Ethical approval for the survey was granted by the University of Central Lancashire Ethics Committee, and all analyses were conducted in accordance with relevant guidelines and regulations.

Briefly, all serving UK firefighters were eligible to take part in the survey and were recruited to participate via email with the aid of the UK’s Fire Brigades Union. Informed consent was obtained from all participants.

The survey consisted of 64 questions covering five key topics: demographics, PPE, contamination, attitudes, awareness and training, and health (see Supplemental File S1). The responses of a total of 10,649 firefighters were included for analysis, representing approximately 24% of the UK’s total firefighter workforce^8^.

### Cancer

Over 4% of serving firefighters (n = 441), who responded to the questionnaire, indicated that they had received a cancer diagnosis. 85% (n = 373) of those firefighters provided further details on their diagnosis. To determine whether diagnoses might be linked to occupational exposure, these firefighters were asked to indicate how many years they had served in the Fire and Rescue Service prior to diagnosis. Any firefighters who indicated their diagnosis was received before joining the Fire and Rescue Service (n = 17) were excluded from further analysis. Similarly, firefighters who did not wish to provide an answer to this question (n = 49) were also excluded from further analysis. Thus, any subsequent analyses on the relationship between cancer and exposure to fire toxins are based on the 307 firefighters who confirmed their cancer diagnosis was received after they joined the Fire and Rescue Service.

### Analysis

Firefighters’ cancer diagnosis status was cross tabulated with several proxies of contaminant exposure. The proportion of firefighters with a cancer diagnosis in each exposure category was then compared. For example, 17 flexi-duty firefighters were diagnosed with cancer out of a total of 335, thus the proportion of flexi-duty firefighters with cancer is reported as 5%. Odds ratios (OR), with 95% confidence intervals, were used to assess the statistical significance of differences between more/less exposed groups.

Multiple logistic regression was used to account for the confounding effects of other (i.e. non-contaminant) cancer risk factors for which there is evidence in the scientific literature (e.g. smoking^16^, excessive alcohol consumption^17^ etc.). Analyses were conducted using Statistical Package for Social Sciences (SPSS) version 28.0.1.1 and the statsmodels module for Python^18^. A step-wise backward variable selection method was used and is presented in Supplemental File S2. Initial modelling included all well-known, non-contaminant cancer risk factors assessed in the survey i.e. sunbathing, excessive drinking, smoking, diabetes, high blood pressure, problems sleeping, and age (Supplementary File S2). However, age was the only independent variable in the final analysis model found to have a significant positive relationship with cancer incidence in UK Fire and Rescue Services (Supplementary File S2). Therefore, all reported odds ratios are adjusted for age (unless otherwise stated).

## Results and analysis

Participant demographics were analysed and presented in Wolffe et al. (2023)^8^, and were not found to significantly differ from the English firefighter population^19^ with respect to sex (*p* > 0.05), but appeared to under-represent younger age categories, retained firefighters, and those belonging to an ethnic minority (*p* < 0.05).

Around three percent of firefighters (n = 307) indicated that they had received a cancer diagnosis after joining the Fire and Rescue Service. The modal age of diagnosis was 45–49 (28% firefighters with cancer).

**Figure 1:**
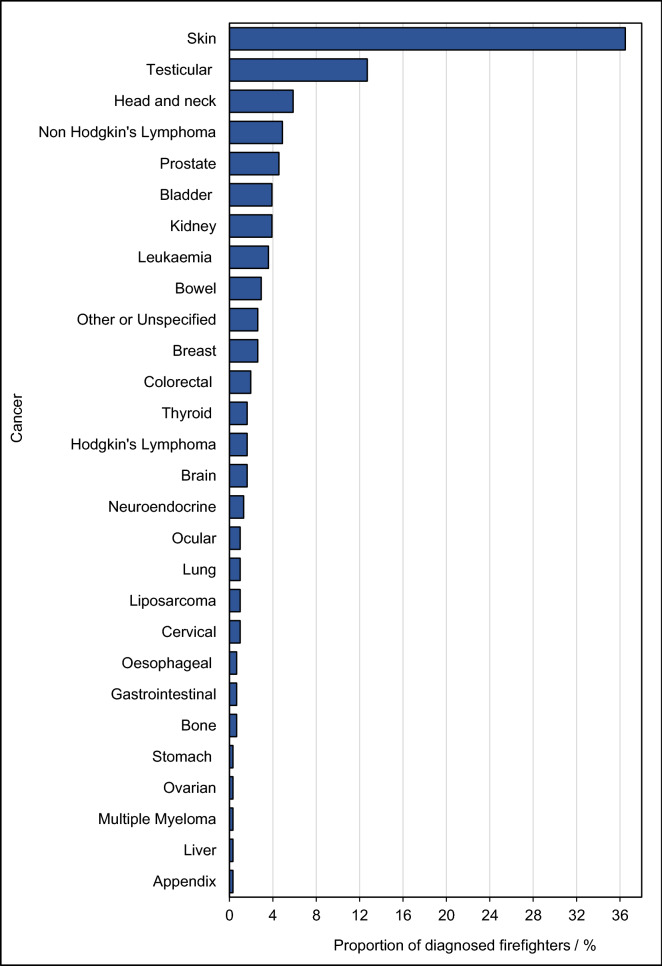
**Range and frequency of cancers among diagnosed firefighters in the UK Fire and Rescue Service.** Prevalence of specific cancers among firefighters who received a diagnosis after joining the Fire and Rescue Service (i.e. a % of 307). Cancers were reported as free text and manually coded for analysis.

Twenty-eight different cancers were coded from the free-text responses of those diagnosed after joining the Fire and Rescue Service. Figure 1 displays the range and frequency of these cancers. Skin cancer was by far the most prevalent cancer reported (36% firefighters with cancer).

### Demographics and cancer

Participant demographics were compared to English FRS statistics^20^ in Wolffe et al. (2023)^8^, and were in broad agreement.

**Figure 2:**
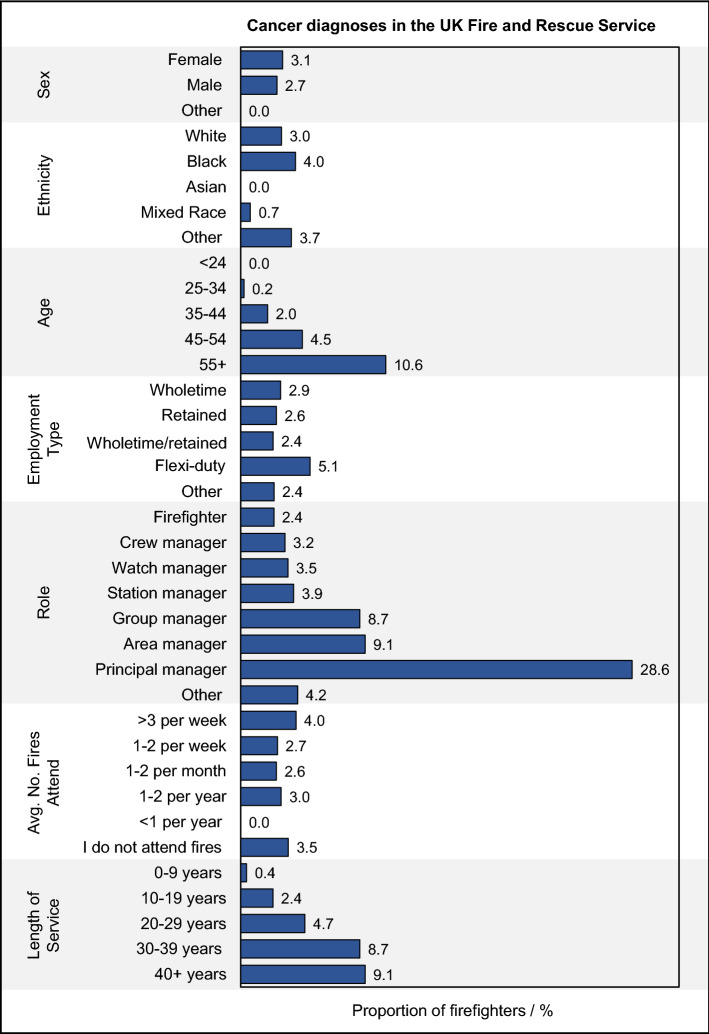
**Demographics and Cancer Prevalence in the UK Fire and Rescue Service.** The proportion of firefighters with a cancer diagnosis in each demographic category is presented (i.e. a percentage of the total number of female firefighters in the survey, male firefighters in the survey, etc.). Note that the relatively small sample size of certain demographic groups (e.g. female firefighters) mean the above results should be interpreted cautiously (see Wolffe et al., 2023 for demographic proportions). Also note that wholetime firefighters work full-time contracted hours/shift patterns. Retained firefighters do not have contracted hours, but are kept on a paid “retainer” contract, remaining on-call for emergencies. Wholetime/ retained firefighters represent wholetime firefighters who work a second retained contract outside of wholetime hours. Flexi-duty firefighters work full-time contracted hours in a more flexible shift pattern.

The proportion of firefighters with a cancer diagnosis increased with age, length of service and seniority of role (Fig. 2). When adjusting for age, managerial firefighters (i.e. crew, watch, station, group, area, and principal managers combined) were not found to be any more likely to develop cancer than non-managerial firefighters (OR = 1.1, 0.8–1.3). However, firefighters who had served 15 years or longer were 1.7 times more likely to develop cancer than those who had served less than 15 years (OR = 1.7, 1.2–2.5).

Given the strong contribution of age to cancer diagnosis status^21^, a crude age-specific cancer rate was used to better compare the prevalence of cancer among surveyed firefighters to that of the general UK population (Table 1). Crude age-specific rates are calculated by dividing the number of firefighters in each age bracket with a cancer diagnosis by the total number of surveyed firefighters and multiplying by 100,000. It is worth noting that the relatively small sample size for female firefighters may skew results.

**Table 1:** Crude, age-specific rate (per 100, 000 people) of cancer in surveyed firefighters compared to the population of England in 2017^22^.

Age at cancer diagnosis	Crude age-specific rate of cancer diagnosis (per 100 000 people)		
	Male (M)/Female (F)	Surveyed firefighters	English population (for 2017)
20-24	M	37.6	29.7
	F	0	32.3
25-29	M	65.7	46.9
	F	9.4	70.8
30-34	M	262.9	66.1
	F	18.8	117.9
35-39	M	375.6	88.8
	F	18.8	168.6
40-44	M	441.4	119.5
	F	75.1	257.4
45-49	M	610.4	208.5
	F	65.7	410.0
50-54	M	394.4	384.0
	F	9.4	566.6
55-59	M	47.0	676.4
	F	0	724.4
60-64	M	37.6	1127.1
	F	0	959.3

Flexi-duty firefighters appeared to be nearly twice as likely to develop cancer than those on all other contract types combined (crude OR = 1.9, 1.1–3.1). However, many surveyed flexi-duty firefighters were over 50 years old (47%). Consequently, they were not found to have a significantly increased cancer odds ratio when adjusting for their age (OR = 1.2, 0.8–2.1).

The geographic distribution of cancer diagnoses is presented in Supplementary File S2 (Fig. S9).

### Firefighters’ health and lifestyle

Firefighters were asked to provide information on several lifestyle factors with known links to chronic health conditions such as cancer, i.e. exercise habits, alcohol consumption, smoking and sunbathing habits (see Supplemental Files S1-S2).

Firefighters in several “higher risk” lifestyle categories did not have a statistically significant increased cancer odds ratio (i.e. those that exercise 2 h or less per week compared to those who exercise more frequently, those that sunbathe/use sunbeds compared to those who never sunbathe/use sunbeds, those that drink 15 units or more of alcohol per week compared to those who drink fewer than 15 units per week, and those who smoke compared to those who do not smoke).

Annual fitness tests were available for just over half (52%) of all surveyed firefighters, with the exception of breathing apparatus (BA) instructors, who indicated via free-text that these tests were available on a 6-monthly basis.

**Figure 3:**
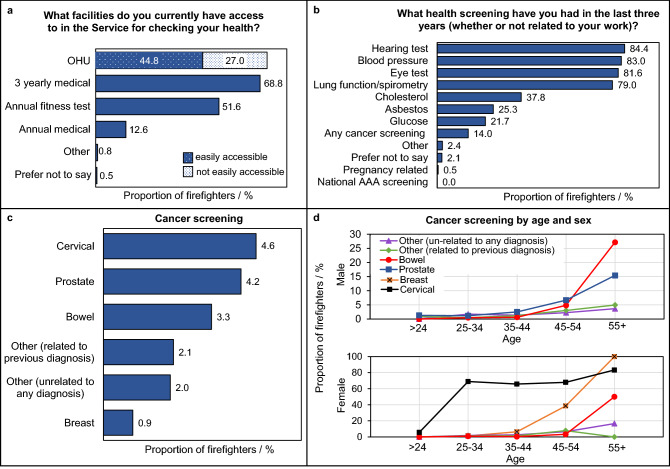
**Health Screening in UK Fire and Rescue Services.** The proportion of total surveyed firefighters who (**a**) have access to health screening facilities within their Service, (**b**) received specific health screening/tests over the last three years, and (**c**) received specific cancer screening over the last three years. Note firefighters can choose more than one type of screening and screening facility. (**d**) The proportion of male or female firefighters in each age bracket who received specific cancer screenings. AAA = Abdominal Aortic Aneurysm, OHU = Occupational Health Unit.

Around 69% of firefighters indicated that they had access to a regular, 3-yearly medical screening for their health. A smaller proportion of firefighters (13%) indicated access to an annual medical health screening (Fig. 3). An exception was for BA instructors, who were provided with a 6-monthly medical screening (data not presented).

Access to an Occupational Health Unit (OHU) was available to a majority of surveyed firefighters (72%), although 27% of firefighters said that this facility was not readily accessible. No clear trend was observed for FRS location/size and access to an OHU (Supplementary File S2, Fig. S7). A similar proportion of managerial (76%) and non-managerial (70%) firefighters had access to an OHU (Supplementary File S2, Fig. S8), although managers were 1.3 times more likely to have access to an OHU when compared to non-managerial firefighters (crude OR = 1.3, 1.2–1.5).

Figure 3 presents the most common health-related screening/tests firefighters had received over the past three years. These were: hearing tests (84%); blood pressure tests (83%); eye tests (82%); and lung function/spirometry (79%). Any type of screening for cancer (whether routine or diagnostic) was received by 14% of all surveyed firefighters. The majority of firefighters who selected the “other” option indicated that they had received none of the listed health screenings (equivalent to 1.2% of all surveyed firefighters).

Cancer screenings were generally positively correlated with age, with the proportion of firefighters receiving screening increasing for older age brackets (Fig. 3).

As well as cancer, firefighters were asked to indicate whether they have had other health conditions such as: problems sleeping, high blood pressure, or diabetes; all of which increase cancer risk. However, no significant association between these conditions and cancer incidence was found for surveyed firefighters after adjusting for age (Supplemental File S2).

### Occupational exposure to toxins and cancer

#### Exposure to fire contaminants at/during the fire incident

A larger proportion of firefighters who indicated that they *often* attend incidents without wearing respiratory protective equipment (RPE) were diagnosed with cancer after joining the Service compared to those who *never* attend incidents without wearing RPE (3.4% versus 2.9%). However, the difference between these two groups was not found to be statistically significant (OR = 1.2, 0.8–1.7)). Similarly, no statistical significance was found for firefighters who only sometimes (versus never) attend fire incidents without RPE (OR = 0.9, 0.7–1.3).

Interestingly, a statistically significantly larger proportion (*p* < 0.05) of cancer diagnoses was found for those firefighters who preferred not to say if they attended fire incidents without wearing RPE (5.7%) when compared to firefighters who always wear RPE. In fact, those who preferred not to say were more than twice as likely to indicate they had been diagnosed with cancer compared to those who *never* attend without RPE (crude OR = 2.2, 1.0–4.9).

It should be noted that RPE covers a wide range of equipment, providing different levels of protection. For example, breathing apparatus is designed to protect the wearer from inhaling toxic gases/particulates, whereas disposable dust masks only offer protection from larger particles (i.e. not from inhalation of toxic gases). Thus, the frequency with which surveyed firefighters attend incidents with/without RPE may not accurately reflect potentially significant differences in exposure to contaminants.

#### Personal contamination

**Figure 4:**
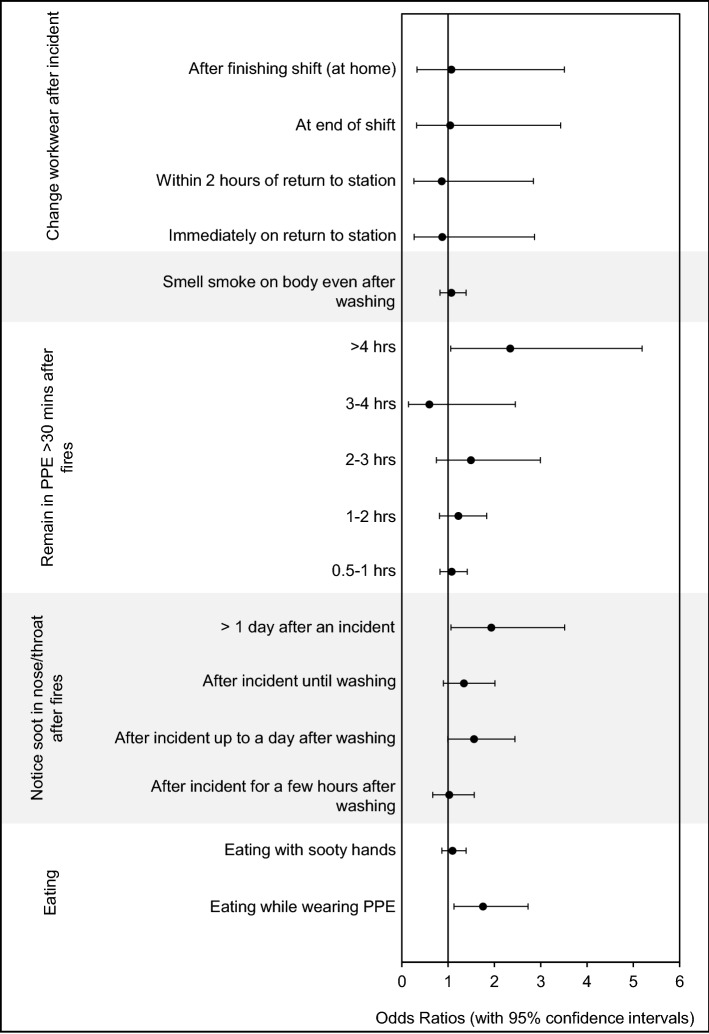
**Personal contamination and cancer odds ratios for UK Firefighters.** Odds ratios (with 95% confidence intervals) were adjusted for age.

Several measures of personal contamination were significantly associated with an increased cancer odds ratio, i.e. eating while wearing PPE (OR = 1.8, 1.2–2.7), noticing soot in the nose/throat for an extended time after attending an incident (OR = 2.0, 1.1–3.5), and staying in PPE for more than four hours after an incident (OR = 2.3, 1.1–5.2). ORs are presented in Fig. 4.

No clear association was found between cancer diagnosis and firefighters’ subjective ratings of feeling contaminated (Supplementary File S2, Fig. S10). However, a clear trend in which areas firefighters felt were most contaminated after fire incidents was found. Face, neck, hands and hair were all consistently rated as feeling more highly contaminated than arms, legs, and trunk.

#### Contaminated PPE and cancer

**Figure 5:**
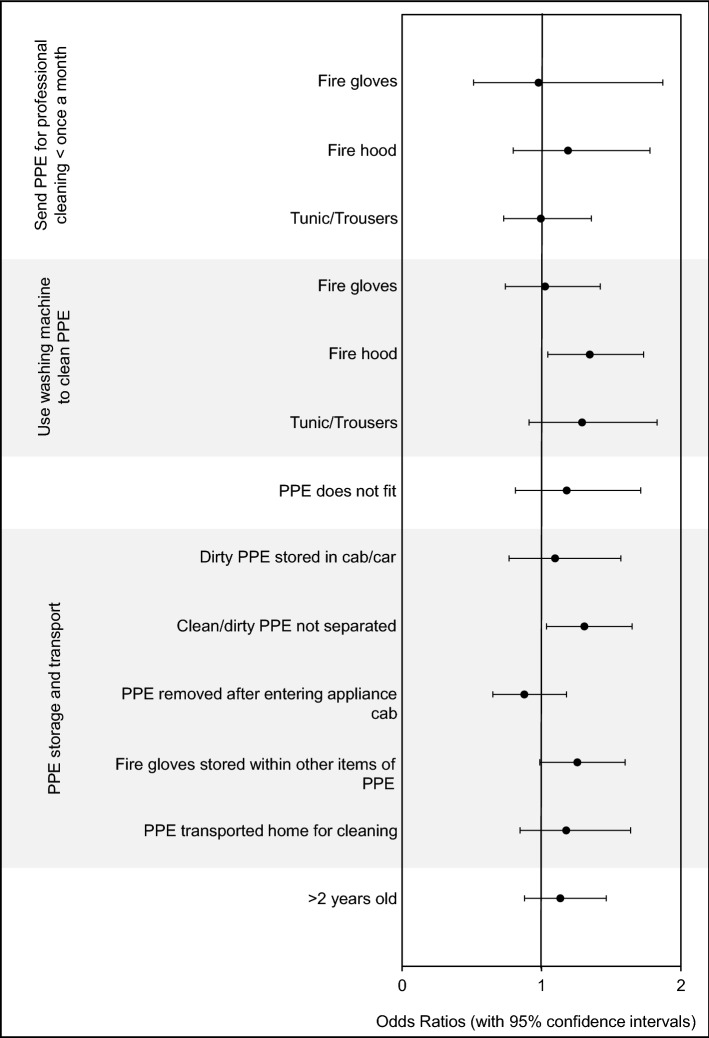
**PPE contamination and cancer odds ratios for UK firefighters.** Odds ratios (with 95% confidence intervals) were adjusted for age.

Almost all proxies of contaminant exposure from PPE were associated with an increased odds ratio of developing cancer among surveyed firefighters (Fig. 5). However, this increase was only statistically significant for failing to store clean and dirty PPE separately (OR = 1.3, 1.0–1.7), and for using a washing machine to clean contaminated fire hoods (OR = 1.3, 1.0–1.7), with storing fire gloves within other items of PPE (i.e. within helmets, boots, and tunic/trouser pockets) near to the threshold of significance (OR = 1.3, 1.0–1.6).

PPE fit did not appear to significantly influence the proportion of firefighters with a cancer diagnosis (OR = 1.2, 0.8–1.7). Roughly equal proportions of firefighters (around 3%) in each PPE fit category received a cancer diagnosis (i.e. PPE fits—2.9%, PPE does not fit—3.1%, prefer not to say—2.9%). Similarly, a roughly equal proportion of firefighters were diagnosed with cancer among those issued with personal-use PPE (2.9%) and those issued with pooled-stock PPE (3.2%, OR = 1.2, 0.9–1.6 for pooled vs personal-use PPE).

### Contamination at the workplace and cancer

**Figure 6:**
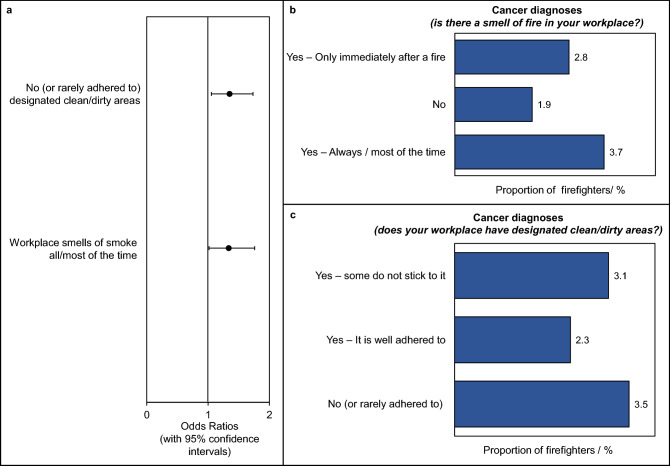
**Workplace contamination and cancer diagnosis in the UK Fire and Rescue Service.** (**a**) Cancer odds ratios for measures of workplace contamination. (**b**), (**c**) The proportion of firefighters in each workplace contamination category who received a cancer diagnosis. Odds ratios (with 95% confidence intervals) were adjusted for age.

Two survey questions asked firefighters about fire contamination at their station, assessing whether workplaces smelt of fire, and whether workplaces had a system for designating separate clean and dirty areas. For both questions, the proportion of firefighters with cancer was greater for those who indicated workplace contamination compared to those who did not (Fig. 6B, C). Both measures of workplace contamination were significantly associated with an increased odds ratio of developing cancer: OR = 1.3 (1.0–1.8) for fire smell in the workplace, and OR = 1.4 (1.1–1.7) for lack of (or unadhered to) clean/dirty areas (Fig. 6A).

Interestingly, smell of fire in the workplace was reported by a majority of surveyed firefighters (87%) regardless of whether those firefighters had designated clean/dirty areas within their workplaces. However, it was found that firefighters whose stations did not have designated clean/dirty areas were almost twice as likely to report a smell of fire in the workplace compared to those working in stations *with* designated clean/dirty areas (crude OR = 1.8, 1.5–2.0).

### Attitudes, awareness and training

**Figure 7:**
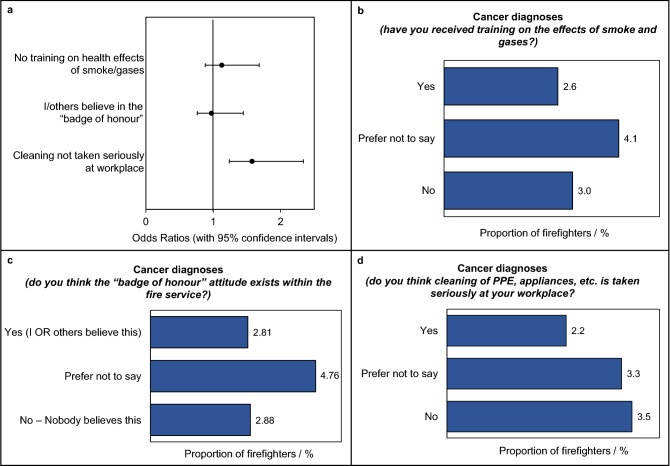
**Attitudes, awareness and training and cancer diagnosis in the UK Fire and Rescue Service.** (**a**) Cancer odds ratios for measures of contaminant exposure due to attitudes/training. (**b**), (**c**), (**d**) The proportion of firefighters in each training/attitude category who have received a cancer diagnosis. Odds ratios (with 95% confidence intervals) were adjusted for age.

Firefighters who believed that cleaning was not taken seriously at their workplace had a significantly higher cancer odds ratio compared to those who felt that cleaning was taken seriously (OR = 1.5, 1.2–2.0), Fig. 7A. Figure 7D illustrates the larger proportion of firefighters diagnosed with cancer among those who believe that cleaning is not taken seriously. Not receiving training on fire toxins and their health outcomes was also associated with an increased odds ratio of developing cancer, although this was not found to be statistically significant (OR = 1.2, 0.9–1.5), Fig. 7A (see similar proportions of cancer diagnoses among these two groups in Fig. 7B). Similarly, believing in the “badge of honour” was almost equivalent to not believing in the “badge of honour” in terms of reporting cancer diagnoses (OR = 1.0, 0.8–1.2, Fig. 7A, C).

## Discussion

### Prevalence of cancer in UK firefighters

The survey found that only 14% of firefighters had received screening for cancer in the last three years, and that this was mostly the age-related screening offered through the National Health Service (NHS) to all UK citizens. Only 2% of firefighters had received cancer screening which was not a part of this NHS provision, and was also not related to a previous cancer diagnosis (Fig. 3).

Skin cancer was most prevalent amongst firefighters diagnosed with cancer after joining the Fire and Rescue Service (Fig. 1), mirroring international findings^23–26^. The most common cause of skin cancer is exposure to UV radiation^27^. Although the survey did not account for time spent working outdoors without protective clothing, firefighters who actively sunbathe/use sunbeds were not found to be at a significantly increased risk of cancer compared to those that never sunbathe (OR = 0.8, 0.6–1.0). This suggests that firefighters’ occupational exposures may plausibly account for the high incidence of skin cancer, likely due to dermal exposure to carcinogenic contaminants^3,4^.

Skin cancers are not routinely screened as these cancers can usually be readily identified by patients at an early stage^28^. Given firefighters increased risk of skin cancers, training and/or awareness campaigns may be beneficial in helping them to identify the early signs of such cancers^29^.

Firefighters were found to suffer cancer at a higher age-specific rate than the general UK population (Table 1). However, there is currently no formal provision for preventatively screening firefighters for cancer within the UK Fire and Rescue Service. The successful treatment of cancer, and subsequent cancer survival rates, are highly dependent on early detection^29^. Thus, given the higher rate of cancer in younger firefighters within the survey (Table 1), additional screening at earlier stages of life may be required to safeguard firefighters’ health.

### Firefighters exposure through contaminated PPE

Following fire incident attendance, firefighters’ PPE may become heavily contaminated with components of fire smoke. This has significant implications for firefighters who remain in contaminated PPE for considerable lengths of time after attending an incident, as it increases the window of exposure to fire toxins, via both inhalation (while toxins continue to off-gas) and dermal (as toxins permeate through fabric to the skin^3,6^) routes.

In fact, the survey found that firefighters who remain in PPE for more than four hours after attending a fire are over twice as likely to develop cancer than those who remain in PPE for 30 min or less after an incident (OR = 2.3, 1.1–5.2). If not effectively removed, contaminants will remain on PPE where they can continue to accumulate, posing a long-term exposure risk. Removing contaminants from PPE and firefighters’ skin (e.g. through washing after an incident) as soon as possible is therefore vital for reducing both acute and chronic dermal exposure.

### Cross contamination

While decontamination plays a major part in reducing exposure to harmful contaminants, emerging research indicates that, without careful consideration of the techniques and procedures used, cleaning/decontamination efforts can contribute to harmful cross-contamination^30^. Cross contamination occurs when otherwise clean items come into direct or indirect contact with dirty/contaminated items^30^. A key example of cross-contamination arising through poorly controlled cleaning procedures is the use of on-site washing machines to launder both contaminated and non-contaminated items^30–32^. Mayer et al. (2019) identified significant cross contamination when laundering fire hoods^30^, supporting findings from the survey, which revealed that the use of washing machines to launder fire hoods was associated with a significantly increased odds ratio of developing cancer (OR = 1.3, 1.0–1.7). It should also be noted that 12% of surveyed firefighters indicated that they take their PPE home to launder. These findings are of concern to firefighters’ family members, who may be exposed to contaminants through potential cross-contamination in home washing machines.

Other potential sources of cross contamination found to significantly increase cancer odds ratios were failing to store clean and dirty PPE separately (OR = 1.3, 1.0–1.7), and failing to designate separate clean and dirty “zones” within the fire station (OR = 1.4. 1.1–1.7).

Storing fire gloves within other items of PPE, e.g. within helmets and boots, was also associated with an increased cancer odds ratio (OR = 1.3, 1.0–1.6), although at the threshold of significance. This practice is of particular concern given that 71% of surveyed firefighters indicated that their fire gloves were *never* sent for professional cleaning, and that 20% indicated that their fire gloves were never cleaned at all^8^.

### Minimising exposures to fire toxins: regulations and accountability

As well as effective PPE decontamination and storage practices, UK Fire and Rescue Services should implement rigorous procedures which control and minimise firefighters’ exposures to fire toxins, in order to reduce cancer risk. Survey results suggest a link between lack of such procedures (e.g. cleaning not taken seriously in the workplace, absence of designated clean/dirty areas) and increased cancer odds ratios (Fig. 6).

However, workplace decontamination procedures are only effective with full compliance, as just one non-compliant individual can cause cross contamination which adversely impacts compliant individuals. The survey found several measures of personal contamination were associated with an increased cancer odds ratio, e.g. noticing soot in the nose/throat (OR = 2.0, 1.1–3.5), eating while wearing PPE (OR = 1.8, 1.2–2.7), staying in PPE for an extended amount of time after an incident (OR = 2.3, 1.1–5.2). Holding firefighters accountable to personal decontamination procedures in the first instance (e.g. changing out of PPE as soon as possible after an incident, showering on return to the station, avoiding eating while wearing PPE etc.^9^) may help promote adherence to community-level decontamination measures, such as those implemented in the wider workplace.

### Culture and awareness

Tackling the “badge of honour” attitude within the UK Fire and Rescue Service may also help to promote adherence to decontamination procedures. This attitude refers to the outdated belief that heavily soiled/contaminated PPE should be celebrated as a symbol of hard-work and dedication among firefighters^15^. The survey found that 16% of firefighters still hold this belief, while 46% believe that others do. Although not significantly associated with an increased risk of cancer, this attitude was positively associated with proxies of fire toxin exposure which were found to be significantly associated with cancer e.g. eating while wearing PPE and staying in PPE for an extended amount of time after an incident (explored further in Wolffe et al. 2023^15^).

Similarly, training on fire contaminants and their health outcomes may also help promote compliance with decontamination procedures. However, whether firefighters had received such training did not appear to significantly increase their odds ratio of developing cancer.

Additionally, finding that a significantly higher proportion of firefighters diagnosed with cancer preferred not to say if they attend fires without RPE (compared to those who always wore RPE) hints at a potential disconnect between firefighters’ awareness and uptake of contaminant control measures. These firefighters may plausibly “prefer not to say” as a means of avoiding accountability for participating in a practice which they know to be a risk to their health and safety. Such disconnect has been found in a surveyed population of US firefighters, and in other occupational settings^33–35^. This may imply that, while potentially beneficial for improving compliance and awareness, one of the most effective measures for reducing cancer incidence in UK firefighters is simply to have and strictly enforce decontamination procedures/policy in the first instance (combined with regular training and inspection).

## Limitations and future work

Analysis of the UK Firefighter Contamination survey revealed several significant associations between exposures to fire contaminants and cancer diagnosis in the UK Fire and Rescue Service. These findings are valuable for the development of effective decontamination procedures/policy, and/or for targeting interventions designed to reduce these exposures and the rate of cancer among firefighters.

Several of the survey’s limitations are outlined in Wolffe et al. 2023^8^ and include potential participation bias; whereby firefighters with cancer or grievances against the UK Fire and Rescue Service may have been more likely to complete the survey than those without cancer/grievances.

Participation bias may also be more pronounced in demographic groups with a relatively small sample size, e.g. female firefighters, and those in senior roles. Conversely, the small sample size of these groups may mean that their demographic-specific findings are underpowered.

Survey questions did not consider all exposures a firefighter has received throughout their career, only assessing firefighters’ current practice/s. Given that repeat (chronic) exposures to carcinogenic contaminants increase the likelihood of developing cancer, results of the survey analysis may be weakened by its failure to account for firefighters’ past exposures.

Cancer is a complex disease, with a variety of inherent risk factors and modes of progression. The survey was also limited in that it did not include all risk factors (e.g. family history, exposure to UV radiation etc.). Study of a wider variety of inherent cancer risk-factors may provide a more accurate view of cancer risk in the UK Fire and Rescue Service.

## Conclusion

Serving UK firefighters were found to have much higher cancer incidence rates than the general population of the same age. Contaminated PPE is a potentially significant source of dermal exposure which increases the risk of developing cancer. Failing to implement procedures which control cross-contamination and/or firefighters’ personal contamination were also associated with a significantly increased likelihood of developing cancer.

Survey results identified several areas for immediate intervention. These include procedures/policy which target cross-contamination e.g. keeping clean/dirty PPE separately, designating specific clean/dirty zones within the station etc. Interventions which target personal contamination (e.g. avoiding eating while wearing PPE, changing PPE/workwear as soon as possible after an incident etc.) should also be considered as both a means of reducing the incidence of cancer in firefighters, and of promoting compliance with less tangible, (community-level) workplace decontamination practices.

It should also be noted that the survey uncovered considerable variation among firefighters in the same demographic, fire exposure, and health outcome categories. For example, firefighters from the same geographic region indicated access to different decontamination facilities/procedures etc. Similarly, surveyed firefighters indicated (via free text) use of different respiratory protection equipment, from disposable dust masks to breathing apparatus^8^.

Overall, these findings reflect the need for better standardisation of decontamination procedures/policies and facilities within the UK Fire and Rescue Services. Equally important and essential for safeguarding firefighters’ health, is the provision of health screening, regularly, and preferably at earlier stages of life.

## Supplementary Information

Supplementary Information 1.

Supplementary Information 2.

## Data Availability

The datasets generated and/or analysed during the current study are available from the corresponding author on reasonable request.
