# Supplementary material for: Cancer incidence amongst UK firefighters
Source: Sci Rep. 2023 Jan 10;12:22072. doi: 10.1038/s41598-022-24410-3 (PMC9831995; doi:10.1038/s41598-022-24410-3)
Supplement: Supplementary file 1 — Supplementary Information 1. [file 41598_2022_24410_MOESM1_ESM.docx]

**Supplementary File S1**

Cancer Incidence Amongst UK Firefighters

Taylor A. M. Wolffe^1^, Andrew Robinson^1,2^, Kathryn Dickens^1^, Louis Turrell^1,2^, Anna Clinton^1^, Daniella Maritan-Thomson^1^, Miland Joshi^3^, Anna A. Stec^1,*^

^1^Centre for Fire and Hazards Sciences, University of Central Lancashire, Preston, Lancashire, PR1 2HE, UK

^2^Royal Preston Hospital, Lancashire Teaching Hospitals NHS Foundation Trust, Preston, Lancashire, PR2 9HT, UK

^3^Lancashire Clinical Trials Unit, University of Central Lancashire, Preston, Lancashire, PR1 2HE, UK

^*^Corresponding author: [aastec@uclan.ac.uk](mailto:aastec@uclan.ac.uk)

| **DEMOGRAPHICS** | |
| --- | --- |
| **Survey Question** | **Answers** |
| Are you currently working as a firefighter in the UK? | Yes  No |
| In which Fire and Rescue Service do you work? | Avon  Bedfordshire  Buckinghamshire  Cambridgeshire  Cheshire  Cleveland  Cornwall  Cumbria  Derbyshire  Devon & Somerset  Dorset & Wiltshire  Durham  East Sussex  Essex  Gloucestershire  Greater Manchester  Guernsey  Hampshire  Hereford and Worcester  Hertfordshire  Humberside  Isle of Man  Isle of Wight  Kent  Lancashire  Leicestershire  Lincolnshire  London  Merseyside  Mid and West Wales  Norfolk  North Wales  North Yorkshire  Northamptonshire  Northern Ireland  Northumberland  Nottinghamshire  Oxfordshire  Prefer not to say  Royal Berkshire  Scotland  Shropshire  South Wales  South Yorkshire  Staffordshire  Suffolk  Surrey  Tyne and Wear  Warwickshire  West Midlands  West Sussex  West Yorkshire |
| What is your sex? | Female  Male  Other  Prefer not to say |
| Would you like to provide more information? | *Free text* |
| What is your age? | Under 20  20-24  25-29  30-34  35-39  40-44  45-49  50-54  55-59  60-64  65+  Prefer not to say |
| What is your ethnic group? (Choose one option that best describes your ethnic group or background) | Any other Asian background  Any other Black / African / Caribbean background  Any other ethnic group  Any other Mixed / Multiple ethnic background  Any other White background  Asian / Asian British: Bangladeshi  Asian / Asian British: Chinese  Asian / Asian British: Indian  Asian / Asian British: Pakistani  Black / African / Caribbean / Black British: African  Black / African / Caribbean / Black British: Caribbean  Mixed / Multiple ethnic groups: White and Asian  Mixed / Multiple ethnic groups: White and Black African  Mixed / Multiple ethnic groups: White and Black Caribbean  Other ethnic groups: Arab  Prefer not to say  White: English / Welsh / Scottish / Northern Irish / British  White: Gypsy or Irish Traveller  White: Irish |
| Are you: | Flexi duty  Retained  Wholetime  Wholetime/retained  Other  Prefer not to say |
| What is your role / rank? | Area manager  Crew manager  Firefighter  Group manager  Other  Prefer not to say  Principal manager  Station manager  Watch manager |
| How many years have you been in the fire and rescue service? | 0-4  5-9  10-14  15-19  20-24  25-29  30-34  35-39  40-44  45+  Prefer not to say |
| On average, how many fires do you attend? | 1-2 per month  1-2 per week  1-2 per year  I do not attend fires  Less than 1 per year  More than 3 per week  Prefer not to say |

**Table S1: Demographic survey questions**

| **PPE** | |
| --- | --- |
| **Survey Question** | **Answers** |
| *PPE Age* | |
| How old is your PPE? | Less than a year  1-2 years  3-4 years  5-10 years  11-15 years  More than 15 years  Not sure  Prefer not to say |
| *PPE Decontamination* | |
| We appreciate every call is different, but we would like to know roughly how long you stay in full PPE after attending a fire incident. Please select the top 3 answers that apply to you and rank them 1 (most often) to 3. If only one applies, simply mark that as the most often. | *Respondents able to rank each of the below options 1 (most often), 2 or 3 (least often):*   - Less than 30 minutes - 30 minutes to 1 hour - 1-2 hours - 2-3 hours - 3-4 hours - More than 4 hours - I do not attend fire incidents - Prefer not to say |
| How soon after attending a fire do you typically change your workwear (t-shirt, trousers, etc.) Please select the top 3 answers that apply to you and rank them 1 (most often) to 3. If only one applies, simply mark that as the most often. | *Respondents able to rank each of the below options 1 (most often), 2 or 3 (least often):*   - Before leaving the incident - Immediately on return to the station - Within 2 hours of returning to the station - At the end of my shift - After I have finished my shift (at home) - Prefer not to say   *Options available to retained respondents only:*   - Immediately after returning (to home/work/etc.) - At the end of the day (I keep it on at home/work/etc.) after the call |
| What is the main reason for you cleaning your PPE (or sending it to be cleaned)? (Select all that apply) | - I am instructed to do it - I send it after every fire incident - It is damaged - It is wet - It looks dirty - It looks sooty - It smells - My PPE has never been cleaned - Only when it is contaminated with hazardous materials (asbestos/body fluids/etc.) - Other - Personal hygiene - Prefer not to say |
| If you selected Other, please specify: | *Free text* |
| Do you take any part of your PPE home to clean it? | No  Prefer not to say  Yes |
| How do you transport your PPE home? | I do not take my PPE home  Other  Personal vehicle  Prefer not to say  Service vehicle |
| If you selected Other, please specify: | *Free text* |
| Do you use your home washing machine to wash your PPE? | No  Prefer not to say  Yes |
| How do you normally wash your tunic and trousers? (Select all the apply) | - Brush - Hand wash with detergent/soap - Hand wash with water only - I send it to be professionally cleaned - It is cleaned by a designated person on site - Other - Paper towel - Prefer not to say - These items do not get cleaned - Washing machine |
| If you selected Other, please specify: | *Free text* |
| Approximately how many times per year do you send your tunic and trousers for professional cleaning? | Every month  Every other month  Every other week  Every week  More than once per week  My tunic and trousers are not sent for professional cleaning  Once per year  Prefer not to say  Twice per year |
| How do you normally wash your fire hood? (Select all that apply) | - Brush - Hand wash with detergent/soap - Hand wash with water only - I send it to be professionally cleaned - It is cleaned by a designated person on site - Other - Paper towel - Prefer not to say - This item does not get cleaned - Washing machine |
| If you selected Other, please specify: | *Free text* |
| Approximately how many times per year do you send your fire hood for professional cleaning? | Every month  Every other month  Every other week  Every week  More than once per week  My fire hood is not sent for professional cleaning  Once per year  Prefer not to say  Twice per year |
| How do you normally clean your BA set? (Select all that apply) | - Brush - Hand wash with detergent/soap - Hand wash with water only - I send it to be professionally cleaned - It is cleaned by a designated person on site - Other - Paper towel - Prefer not to say - This item does not get cleaned |
| If you selected Other, please specify: | *Free text* |
| How do you normally wash your fire gloves? (Select all that apply) | - Brush - Hand wash with detergent/soap - Hand wash with water only - I send it to be professionally cleaned - It is cleaned by a designated person on site - Other - Paper towel - Prefer not to say - This item does not get cleaned - Washing machine |
| Approximately how many times per year do you send your fire gloves for professional cleaning? | Every month  Every other month  Every other week  Every week  More than once per week  My fire gloves are not sent for professional cleaning  Once per year  Prefer not to say  Twice per year |
| *PPE Provision* | |
| Do you have PPE for your individual use? | No – I never use PPE  No – I use PPE from pooled stock  Prefer not to say  Yes – I have individually issued PPE |
| How often do you exchange your kit with the pooled stock? | At the end of each watch  I exchange it after every fire incident  Only when it is contaminated with hazardous materials (asbestos, body fluids, etc.)  Other  Personal hygiene  Prefer not to say  When I am instructed to do it  When it is damaged  When it is wet  When it looks dirty  When it looks sooty  When it smells |
| If you selected Other, please specify: | *Free text* |
| Does your PPE always appear clean when you take it from pooled stock? | It has a smell of smoke  It has stains but no smell  No, it appears dirty  Other  Prefer not to say  Yes, it is clean with no smell |
| If you selected Other, please specify: | *Free text* |
| Does your PPE fit well? | No  Prefer not to say  Yes |
| Do you have concerns about the fit of your PPE? | *Free text* |
| Do you ever attend fires (or post fire / turning over / damping down, etc.) without wearing respiratory protective equipment (RPE, eg. BA or half face mask)? | Never  Often  Prefer not to say  Sometimes |
| What are your main reasons for attending fires (or post fire / turning over / damping down, etc.) without wearing respiratory protective equipment (RPE, eg. BA or half face mask)? (Select all that apply) | - Financial constraints - Habit - I am told not to, and I am happy not to wear it - I am told not to, but I would prefer to wear it - I don't have to wear it, so I choose not to - Nobody else wears it - Other - Prefer not to say - The equipment is not available, and I am happy not to wear it - The equipment is not available, but I would prefer to wear it   Time constraints |
| If you selected Other, please specify: | *Free text* |
| *PPE Storage* | |
| Where do you normally store your fire gloves? (Select all that apply) | - In a kit bag with PPE - In a locker - In a specific glove bag - In your boots - In your helmet - On a shelf / bench - Other - Prefer not to say - Tunic or trouser clip/tab (External) - Tunic or trouser pocket |
| If you selected Other, please specify: | *Free text* |
| Where do you normally remove PPE after attending a fire? (Select all that apply) | - At home - I never wear PPE - In the appliance bay at the station - In the clothing room at the station - In the fire appliance - On site before getting into the vehicle (engine/car/etc.) - Other - Prefer not to say |
| If you selected Other, please specify: | *Free text* |
| Where do you store your PPE while travelling from a fire incident? (Select all that apply) | - Designated storage in the appliance - Disposable bag - In the cab - In the car (no bag) - It is collected by someone else from the fire ground - Kit bag - Other - Prefer not to say |
| If you selected Other, please specify: | *Free text* |
| Is clean and dirty PPE stored separately? | No  Prefer not to say  Yes |
| Where is clean PPE normally stored? (Select all that apply) | - Appliance bay - Designated PPE room - Office - Other - PPE locker - Personal locker - Personal vehicle - Prefer not to say |
| If you selected Other, please specify: | *Free text* |
| Where is dirty or "in-use" PPE normally stored? (Select all that apply) | - Appliance bay - Designated PPE room - Office - Other - PPE locker - Personal locker - Personal vehicle - Prefer not to say |
| If you selected Other, please specify: | *Free text* |

**Table S2: PPE survey questions**

| **WORKPLACE CONTAMINATION** | | |
| --- | --- | --- |
| **Survey Question** | **Answers** | **Re-grouping for Crude Odds Ratios** |
| Does your workplace have designated clean / dirty areas? (For example, no equipment in offices/sleeping areas, but it is allowed in appliance bay) | No  Prefer not to say  Yes – But it is rarely/never followed  Yes – It is well adhered to  Yes – some do not stick to it | No (No, Yes – But it is rarely/never followed) vs. Yes (Yes – It is well adhered to, Yes – some do not stick to it) |
| Is there a smell of fire in your workplace? | No  Prefer not to say  Yes – Always / most of the time  Yes – Only immediately after a fire | Yes – Always / most of the time vs. (No, Yes – Only immediately after a fire) |

**Table S3: Workplace contamination survey questions**

| **PERSONAL CONTAMINATION** | | |
| --- | --- | --- |
| **Survey Question** | **Answers** | **Re-grouping for Crude Odds Ratios** |
| Do you ever eat while wearing PPE? | No – Never  Prefer not to say  Yes – Occasionally  Yes – Often | Yes (Yes-Occasionally, Yes-Often) vs. No-Never |
| Do you ever eat with sooty hands? | No – Never  Prefer not to say  Yes – Occasionally  Yes – Often | Yes (Yes-Occasionally, Yes-Often) vs. No-Never |
| On a scale of 1 to 5, can you indicate whether each part of your body feels contaminated with soot / smoke / debris after attending a fire incident or post fire (If you prefer not to say, you don’t need to select any option) | *Respondents able to rank each of the below options 1 (very contaminated), 2, 3 (somewhat contaminated), 4, or 5 (not at all contaminated):*   - Face - Neck - Hair - Hands - Arms - Legs - Trunk | *n/a* |
| Do you ever notice soot in your nose/throat? | No  Prefer not to say  Yes – After an incident, for a few hours after I washed  Yes – After an incident, for up to a day after I washed  Yes – After an incident, until I washed  Yes – For more than a day after an incident | Each “Yes” answer option is in turn compared to “No” |
| How often can you (or others) smell smoke, or the smell of fire, on your body? | After an incident, for a few hours after I washed  After an incident, for up to a day after I washed  After an incident, until I washed  For more than a day after an incident  Never  Prefer not to say | Each time answer option is in turn compared to “No” |
| How much do you feel each part of your body smells like smoke (if at all)? If you prefer not to answer, you do not need to tick any box. | *Respondents able to rank each of the below options 1 (strong smell), 2, 3, 4, or 5 (no smell at all):*   - Hair - Hands - Arms - All over - Can't tell / general smell | *n/a* |

**Table S4: Personal contamination survey questions**

| **ATTITUDE, AWARENESS AND TRAINING** | | |
| --- | --- | --- |
| **Survey Question** | **Answers** | **Re-grouping for Crude Odds Ratios** |
| Do you think the cleaning of PPE, fire appliances, etc. is taken seriously at your station/workplace? | No  Not applicable  Prefer not to say  Yes | No vs. Yes |
| Do you have any comments on how seriously the cleaning of PPE, appliances, etc is taken? Please do not include names of specific people or stations. | *Free text* | *n/a* |
| The "Badge of Honour" attitude refers to firefighters wearing soiled PPE because it might give the impression that they are more qualified or hardworking. Do you think this attitude exists within the fire service? | No – Nobody believes this  Prefer not to say  Yes – I believe this  Yes – Others believe this | Each “Yes” option is in turn compared to “No – Nobody believes this” |
| Have you received training on the effects of smoke and gases, and how these might impact your long-term health? | No - I would find it useful  No - I would not find it useful  Prefer not to say  Yes - I did not find it useful  Yes - I found it useful | No (No – I would find it useful, No- I would not find it useful) vs. Yes (Yes-I did not find it useful, Yes- I found it useful). |
| Do you have any comments about what you think should be done at fire stations/workplaces to protect staff from harmful substances, or what you think is / is not currently done well? | *Free text* | *n/a* |

**Table S5: Attitude, awareness and training survey questions**

| **HEALTH** | |
| --- | --- |
| **Survey Question** | **Answers** |
| Generally, how much do you drink during and immediately after a fire incident? Please select the top 3 answers that apply to you and rank them 1 (most often) to 3. If only one applies, simply mark that as the most often. | *Respondents able to rank each of the below options 1 (most often), 2 or 3 (least often):*   - Less than 0.25L - 0.25 – 0.5L - 0.5-0.75L - 0.75-1L - More than a litre - I do not routinely drink during or straight after a fire - I do not attend fires - Prefer not to say |
| What facilities do you currently have access to in the Service for checking your health? (Select all that apply) | 3 yearly medical Annual fitness tests Annual medical Occupational Health Unit or dedicated medical centre (Easily accessible) Occupational Health Unit or dedicated medical centre (Not easily accessible) Other Prefer not to say |
| What health screening have you had in the last three years (whether or not related to your work)? (Select all that apply) | Asbestos  Blood pressure  Cholesterol  Eye test  Glucose  Hearing test  Lung function/spirometry  National AAA screening (typically offered to men 65+)  National bowel cancer screening (typically offered to men 55+/60+)  National breast screening (typically offered to women aged 50+)  National cervical screening (typically offered to women aged 25-64)  Other  Other cancer screening not related to any diagnosis Other cancer screening related to previous diagnosis  Prefer not to say  Prostate cancer screening  Screening related to pregnancy |
| If you selected Other, please specify: | *Free text* |
| Roughly how many hours of exercise do you do per week? (e.g. gym, running, swimming, sports, etc.) | 0-2 hours per week  2-4 hours per week  4-6 hours per week  6-8 hours per week  8-10 hours per week  10+ hours per week  I do not often exercise  Prefer not to say |
| Approximately how many units of alcohol do you drink per week? | 1-5 units per week  6-10 units per week  11-15 units per week  16-20 units per week  21-25 units per week  26-30 units per week  30+ units per week  I do not drink alcohol  I drink less than once per week  Prefer not to say |
| Do you smoke? | I vape / use an e-cig  No  No - I quit within the last 12 months  Prefer not to say  Yes - 10-20 per day  Yes - 1-10 per day  Yes - 20+ per day  Yes - Less than once per day |
| Approximately how often do you use sunbeds or sunbathe (actively sitting in the sun) (Select all that apply) | - I never sunbathe or use sunbeds - I sunbathe as much as possible in summer - I sunbathe infrequently (e.g. annual holiday) - I use sunbeds every month - I use sunbeds every week - I use sunbeds once or twice per year - Prefer not to say |
| Do you have problems sleeping? (e.g. insomnia, disturbed sleep) | No – Very rarely or never  Prefer not to say  Yes – Often  Yes – Sometimes |
| Do you feel any of the following contribute to your sleep problems? (Select all that apply) | Don’t know  I have a known health complaint that causes it  Mental health  Other  Prefer not to say  Shift work  Workload |
| If you selected Other, please specify: | *Free text* |
| Have you been diagnosed with any blood pressure problems? | No  Prefer not to say  Yes – High blood pressure  Yes – Low blood pressure |
| Do you have diabetes? | No  Prefer not to say  Yes – Type 1  Yes – Type 2 |
| Do you have any mental health conditions? (Select all that apply) | ADHD  ASD (Autism Spectrum Disorder)  Anxiety  Bipolar disorder  Depression  Eating disorder  No known health conditions  OCD  Other  PTSD  Prefer not to say  Schizophrenia |
| If you selected Other, please specify: | *Free text* |
| Have you ever had problems with fertility? (Select all that apply) | - No - I have had no problems trying to conceive - No - I have never tried to conceive - Prefer not to say - Yes – I do not know the reason - Yes – I have a known infertility problem - Yes – My partner has a known infertility problem |
| Have you ever been diagnosed with cancer? | No  Prefer not to say  Yes - I am happy to provide details  Yes - I would prefer not to provide details |
| What type of cancer? | *Free text* |
| Your age at diagnosis: | 0-4  5-9  10-14  15-19  20-24  25-29  30-34  35-39  40-44  45-49  50-54  55-59  60-64  65+ |
| How long you had been in the fire service prior to diagnosis: | 0-4 years  5-9 years  10-14 years  15-19 years  20-24 years  25-29 years  30-34 years  35-39 years  40-44 years  45-49 years  Before joining the fire service |

**Table S6: Health survey questions**
