# Supplementary material for: Cancer incidence amongst UK firefighters
Source: Sci Rep. 2023 Jan 10;12:22072. doi: 10.1038/s41598-022-24410-3 (PMC9831995; doi:10.1038/s41598-022-24410-3)
Supplement: Supplementary file 2 — Supplementary Information 2. [file 41598_2022_24410_MOESM2_ESM.docx]

**Supplemental File S2**

Cancer Incidence Amongst UK Firefighters

Taylor A. M. Wolffe^1^, Andrew Robinson^1,2^, Kathryn Dickens^1^, Louis Turrell^1,2^, Anna Clinton^1^, Daniella Maritan-Thomson^1^, Miland Joshi^3^, Anna A. Stec^1,*^

^1^Centre for Fire and Hazards Sciences, University of Central Lancashire, Preston, Lancashire, PR1 2HE, UK

^2^Royal Preston Hospital, Lancashire Teaching Hospitals NHS Foundation Trust, Preston, Lancashire, PR2 9HT, UK

^3^Lancashire Clinical Trials Unit, University of Central Lancashire, Preston, Lancashire, PR1 2HE, UK

^*^Corresponding author: [aastec@uclan.ac.uk](mailto:aastec@uclan.ac.uk)

**Additional Methodological Detail**

The survey was piloted with a small subset of firefighters, and questions rephrased for clarity according to feedback. Ethical approval for the survey was granted by the University of Central Lancashire Ethics Committee, and all analyses were conducted in accordance with relevant guidelines and regulations.

The survey ran through Jisc software, for a period of 3 months between November 2019 and February 2020. A link to the survey was distributed to participants via email through the Fire Brigades Union (FBU). The survey took approximately 20 minutes to complete and was supported by UK Fire and Rescue Services (FRSs) with respect to allowing firefighters dedicated time within their workday in which to complete it.

Free text answers were manually coded for analysis according to the most commonly appearing themes.

All currently serving UK firefighters were eligible to take part in the survey. Therefore, the first question of the survey, *“Are you currently working as a firefighter in the UK?”*  was used to include/exclude survey responses from analysis. A total of 6 respondents indicated that they were not currently working as firefighters in the UK and were thus excluded from further analysis. A further 4 respondents identified themselves as retired in the free text answers they provided to survey questions and were thus excluded. Four hundred and seventy-one respondents left this initial question blank. Due to the nature of recruitment to the survey (i.e. via email to FBU members), these respondents were assumed to be currently serving UK firefighters and included in subsequent analyses. This left a total of 10,649 included respondents. This figure represents approximately 24% of the UK’s total Firefighter workforce.

**Logistic Regression Model Refinement**

| **Variables in the Equation** | | | | | | | |
| --- | --- | --- | --- | --- | --- | --- | --- |
|  | | B | S.E. | Wald | df | Sig. | Exp(B) |
| Step 1^a^ | Smoker | -.412 | .243 | 2.877 | 1 | .090 | .662 |
|  | Excessive_drinker | .101 | .144 | .494 | 1 | .482 | 1.107 |
|  | Exercise_infrequently | -.168 | .151 | 1.249 | 1 | .264 | .845 |
|  | Sunbather | -.283 | .131 | 4.668 | 1 | .031 | .754 |
|  | Problem_sleeper | .020 | .126 | .025 | 1 | .874 | 1.020 |
|  | High_bp | -.088 | .181 | .239 | 1 | .625 | .915 |
|  | Diabetes | -.188 | .602 | .097 | 1 | .755 | .829 |
|  | Age_pseudo_contin | .530 | .043 | 152.441 | 1 | <.001 | 1.699 |
|  | Constant | -6.738 | .328 | 422.535 | 1 | <.001 | .001 |
| Step 2^a^ | Smoker | -.411 | .243 | 2.866 | 1 | .090 | .663 |
|  | Excessive_drinker | .102 | .144 | .506 | 1 | .477 | 1.108 |
|  | Exercise_infrequently | -.168 | .151 | 1.246 | 1 | .264 | .845 |
|  | Sunbather | -.283 | .131 | 4.658 | 1 | .031 | .754 |
|  | High_bp | -.087 | .181 | .230 | 1 | .632 | .917 |
|  | Diabetes | -.186 | .602 | .096 | 1 | .757 | .830 |
|  | Age_pseudo_contin | .531 | .043 | 152.989 | 1 | <.001 | 1.700 |
|  | Constant | -6.728 | .322 | 437.853 | 1 | <.001 | .001 |
| Step 3^a^ | Smoker | -.413 | .243 | 2.885 | 1 | .089 | .662 |
|  | Excessive_drinker | .103 | .144 | .512 | 1 | .474 | 1.108 |
|  | Exercise_infrequently | -.169 | .151 | 1.259 | 1 | .262 | .844 |
|  | Sunbather | -.283 | .131 | 4.658 | 1 | .031 | .754 |
|  | High_bp | -.092 | .180 | .260 | 1 | .610 | .912 |
|  | Age_pseudo_contin | .530 | .043 | 153.022 | 1 | <.001 | 1.699 |
|  | Constant | -6.725 | .321 | 438.158 | 1 | <.001 | .001 |
| Step 4^a^ | Smoker | -.413 | .243 | 2.883 | 1 | .089 | .662 |
|  | Excessive_drinker | .100 | .144 | .485 | 1 | .486 | 1.105 |
|  | Exercise_infrequently | -.175 | .150 | 1.359 | 1 | .244 | .839 |
|  | Sunbather | -.282 | .131 | 4.650 | 1 | .031 | .754 |
|  | Age_pseudo_contin | .526 | .042 | 156.665 | 1 | <.001 | 1.692 |
|  | Constant | -6.705 | .319 | 442.934 | 1 | <.001 | .001 |
| Step 5^a^ | Smoker | -.405 | .243 | 2.780 | 1 | .095 | .667 |
|  | Exercise_infrequently | -.173 | .150 | 1.332 | 1 | .248 | .841 |
|  | Sunbather | -.279 | .131 | 4.554 | 1 | .033 | .756 |
|  | Age_pseudo_contin | .528 | .042 | 159.299 | 1 | <.001 | 1.695 |
|  | Constant | -6.702 | .318 | 443.869 | 1 | <.001 | .001 |
| Step 6^a^ | Smoker | -.431 | .242 | 3.185 | 1 | .074 | .650 |
|  | Sunbather | -.274 | .131 | 4.395 | 1 | .036 | .760 |
|  | Age_pseudo_contin | .525 | .042 | 158.087 | 1 | <.001 | 1.690 |
|  | Constant | -6.720 | .318 | 447.250 | 1 | <.001 | .001 |
| a. Variable(s) entered on step 1: Smoker, Excessive_drinker, Exercise_infrequently, Sunbather, Problem_sleeper, High_bp, Diabetes, Age_pseudo_contin. | | | | | | | |

**Table S1**: Multiple logistic regression parameters and for known cancer risk factors and backward step-wise variable selection.

Age was the only independent variable found to have a significant (p <0.05) positive association with cancer diagnosis status. Although significant, smoking and sunbathing were both found to have a negative association with cancer amongst survey respondents – contrary to the well established body of literature. This negative association is likely to arise from other factors and/or small sample sizes in the relevant risk factor/cancer diagnosis groups.

Further investigation did not find any significant interaction between age and smoking. However, it should be noted that the surveyed population of (serving) firefighters represent a relatively young workforce – with a normal retirement age of 55 at the time the survey was conducted. Given that cancers most commonly associated with smoking (e.g. lung cancer) tend to appear later in life (most commonly occurring in people over 70) – it is likely that smoking-related cancers may simply not have arisen yet in the surveyed population.

The survey did not ask for sufficient detail on smoking habit e.g. years of smoking, use of chewing tobacco etc. Thus grouping of firefighters as “smokers” (including those who had quit within the last 12 months) and “non-smokers” may be an oversimplification which confounds the seemingly negative association between smoking and cancer.

Potentially wide variation in sunbathing habits which the survey could not account for (e.g. length of sunbathing sessions, whether firefighters wore sunscreen when sunbathing etc.), and the therapeutic effects of sun exposure (e.g. vitamin D synthesis, improved mood, more time spent in fresh outdoor environments etc.), may similarly confound the negative association between sunbathing and cancer.

As removing these variables from the model did not significantly alter the overall results – only age was included in subsequent logistic regression analyses.

|  |  | **Count Cancer** | **Count No Cancer** |
| --- | --- | --- | --- |
| Exercise | Exercise 2 hours or less per week | **62** | **2078** |
|  | Exercise more than 2 hours per week | **244** | **7990** |
| Alcohol consumption | More than 15 units per week | **69** | **1767** |
|  | Less than 15 units per week | **235** | **8196** |
| Sun exposure | Any kind of sunbathing | **213** | **7461** |
|  | Never sunbathe | **93** | **2593** |
| Smoking | Yes (incl if quit in last 12 months) | **24** | **1330** |
|  | No | **282** | **8748** |
| Sleep problems | Rarely or never have problems sleeping | **105** | **3980** |
|  | Problems sleeping | **202** | **6125** |
| Blood pressure | High blood pressure | **41** | **924** |
|  | Low or no blood pressure problems | **261** | **9061** |
| Diabetes | Diabetes | **3** | **80** |
|  | No diabetes | **301** | **9987** |

**Table S2**: Grouping of participants for analysis of potentially confounding health/lifestyle variables. Note that cancer refers to cancer diagnosis *after* joining the Fire and Rescue Service

**Age, Role and Length of Service**

Respondent age was positively correlated with seniority of role (Figure S1), with older age categories (shaded darker in Figure S1A) increasingly represented in more senior roles and younger age categories (shaded lighter in Figure S1A) decreasingly represented. Figure S1B illustrates how the proportion of those who indicated that they held other/senior roles steadily increases with age (Figure S1B) – while the proportion of those indicating that they hold firefighter positions simultaneously decreases with age.


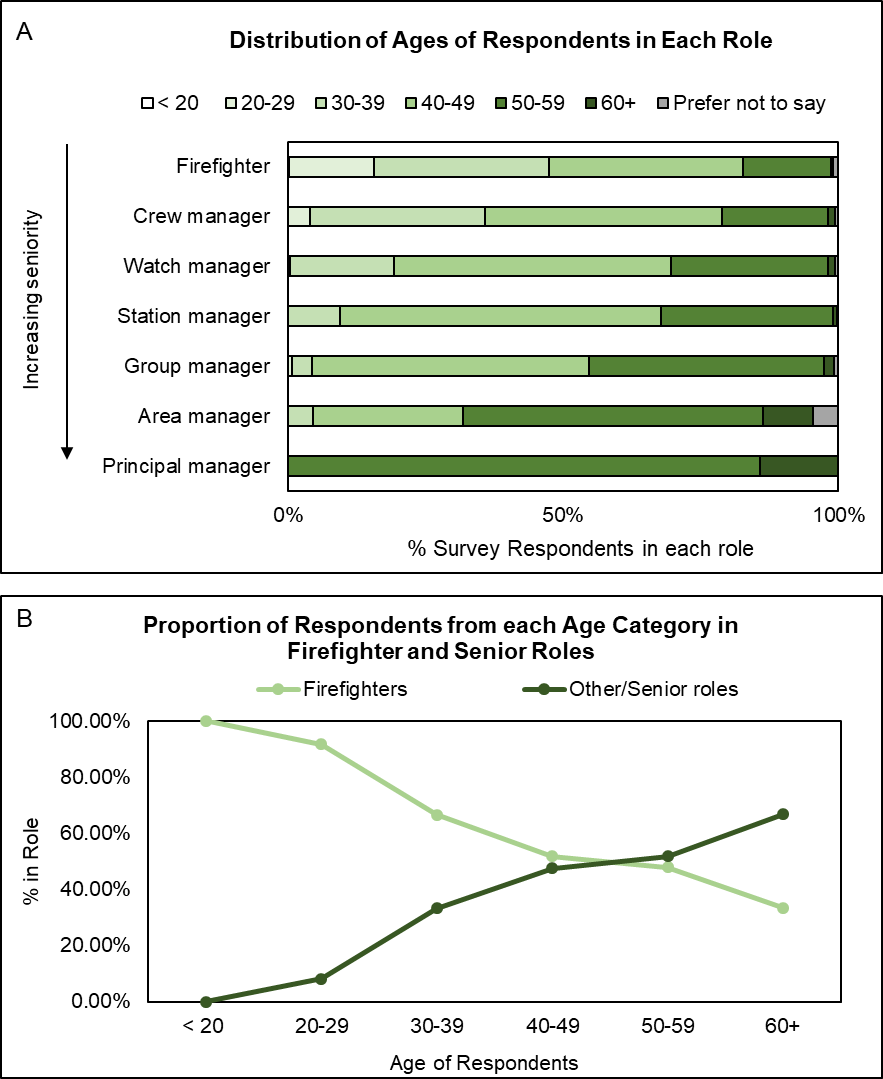

**Figure S1**: A – the proportion of respondents in each role who belong to each of the age categories. B – the proportion of respondents in each age category who hold firefighter and other/senior roles. Both panels demonstrate how age is positively correlated with seniority of role.

Age was also correlated with length of service (see Table S3) – with longer lengths of service becoming more common with increasing age.

| **Age of Respondents** | **Modal Length of Service** |
| --- | --- |
| < 20 | 0-9 years |
| 20-29 | 0-9 years |
| 30-39 | 10-19 years |
| 40-49 | 10-19 years |
| 50-59 | 20-29 years |
| 60+ | 30-39 years |

**Table S3**: The modal length of service recorded for respondents in each age category.

Similarly, length of service and seniority of rank were positively correlated (Figure S2) – with more experienced respondents generally holding more senior roles.


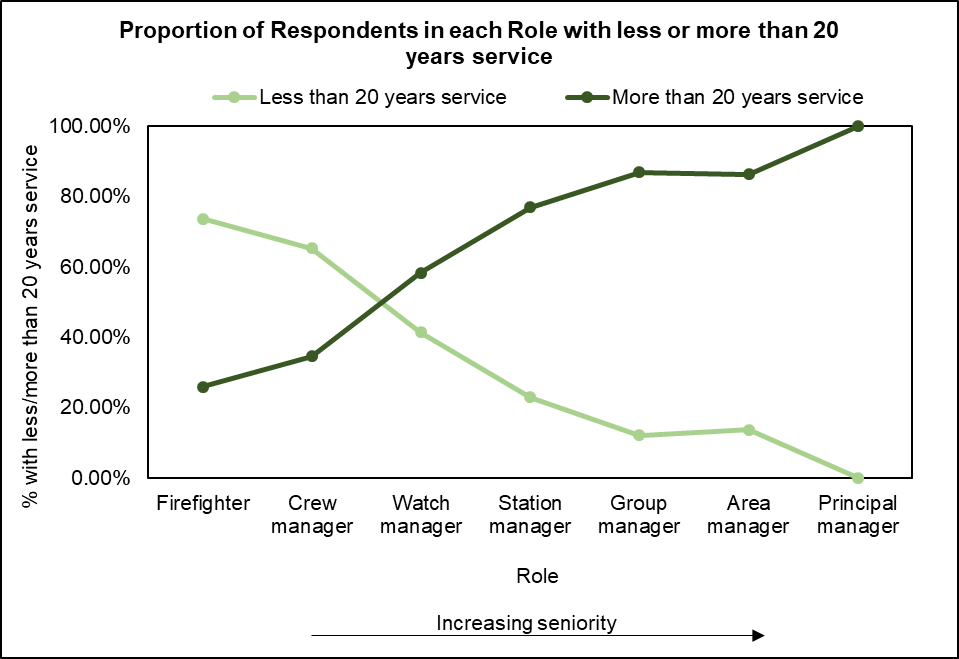


**Figure S2**: The proportion of respondents in each role with less (light green) and more (dark green) than 20 years of service. The proportion of respondents with less than 20 years of service steadily decreases with increasing seniority of rank, while the proportion of respondents with more than 20 years of experience steadily increases.

While changes in role-specific tasks might be expected with increasing age, seniority of role, and length of service – no considerable difference between categories in any of these demographics was found when considering the number of fires survey respondents typically attend (Figure S3), with the majority of respondents in any age, role or length of service category attending 1-2 fires per month or more.


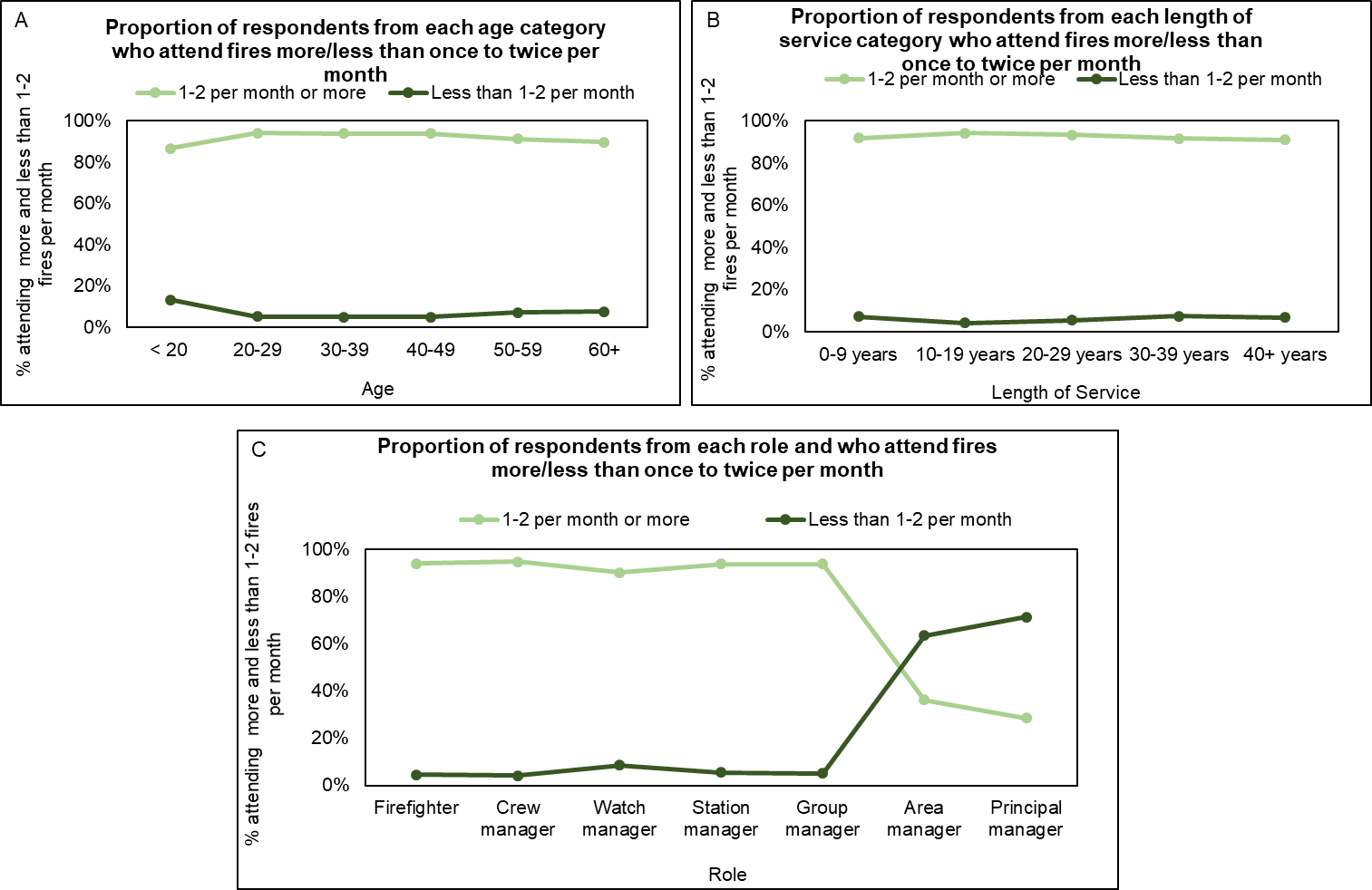


**Figure S3**: Age (A), length of service (B) and role (C) vs. number of fires that respondents typically attend. No considerable differences were observed for any age or length of service category, and for most of the roles – with most respondents attending 1-2 fires per month or more. A higher proportion of area managers and principal managers appear to attend fires less frequently than once to twice per month, but given the relatively small sample size available for these roles (see Table 2) it is difficult to generalise this finding to the wider population of UK firefighters.

**Employment Type**

No clear trend in age distribution was found for employment type (Figure S4) – although older age categories were more significantly represented for other and flexi-duty employment types. However, the *other* employment type also had the largest representation of the youngest age category (<20).

**Figure S4:** Proportion of respondents in each employment type who indicated their age.

The majority of respondents employed on a wholetime/retained, wholetime or retained basis were firefighters (Figure S5) – whereas the majority of respondents employed on a flexi-duty basis were employed in more senior, managerial positions (Figure S5). As might be expected, respondents who indicated their employment type as “other” also represented the highest proportion of respondents indicating their role as “other”.

**Figure S5**: Proportion of respondents in each employment type who indicated their role.

**Health and Lifestyle**


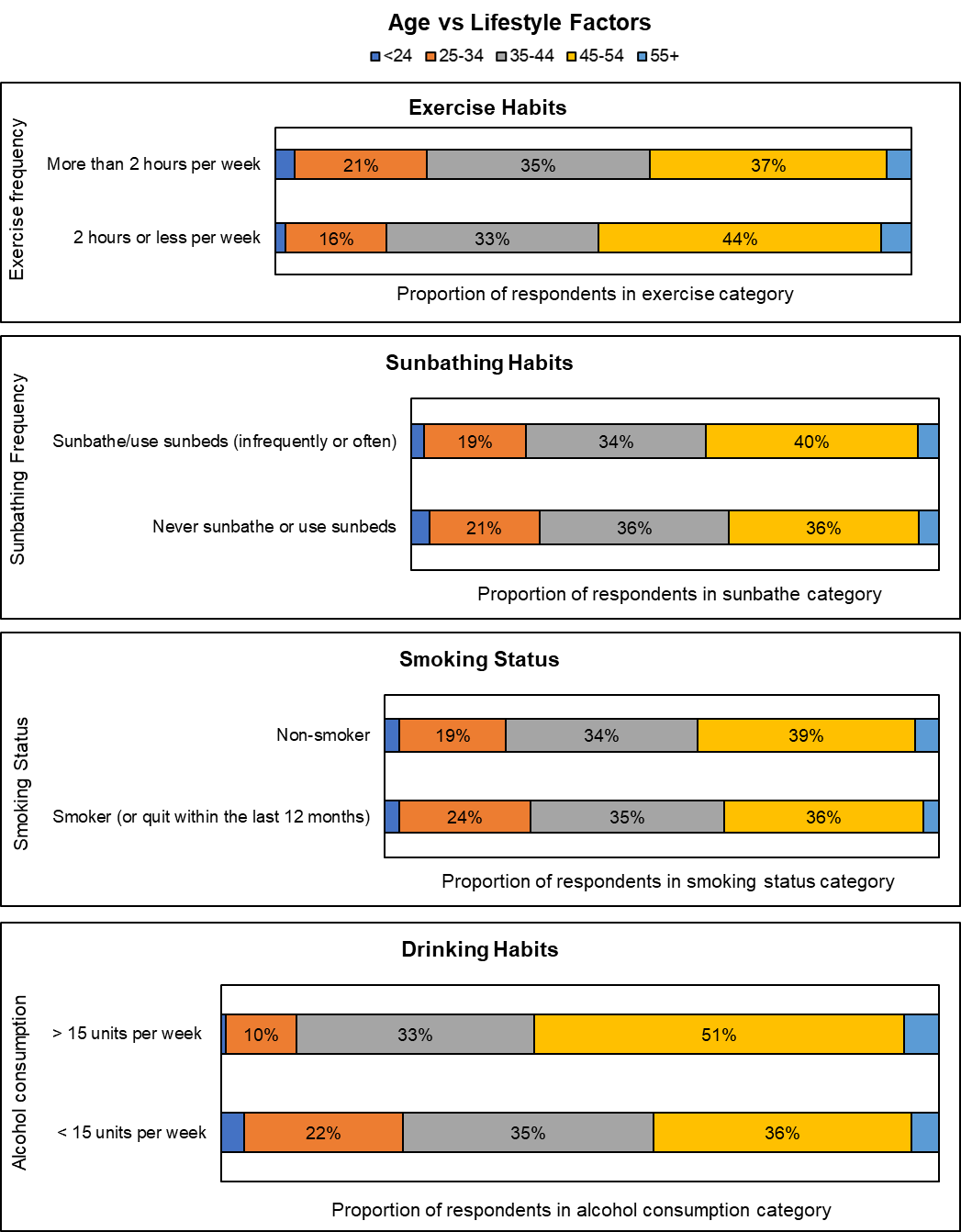


**Figure S6**: Proportion of respondents in each lifestyle category who indicated their age.

**Figure S7**: Access to an Occupational Health Unit within the UK Fire Service


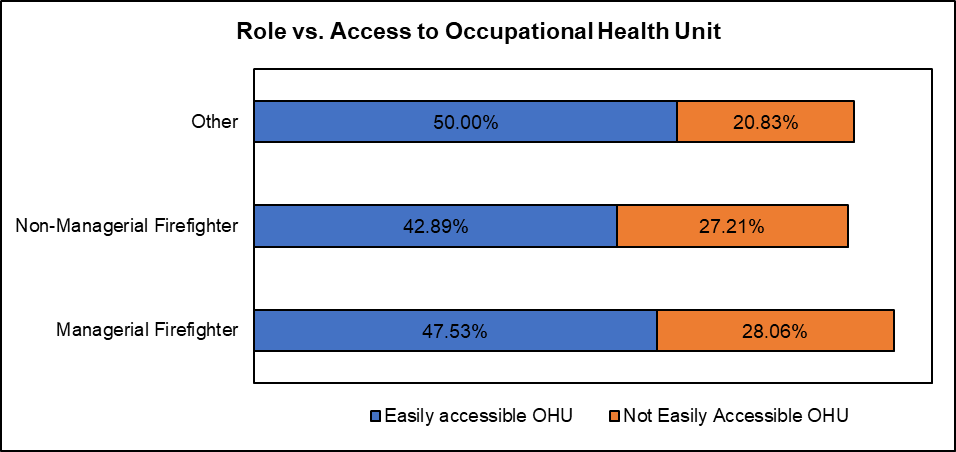


**Figure S8**: Proportion of respondents in each role category who indicated whether they had access to an occupational health unit (OHU).

## **Geographic Distribution**

Respondents with a cancer diagnosis were spread across most geographic regions. However, no respondents were diagnosed with cancer after joining the Service in Cambridgeshire, Gloucestershire, or Guernsey. The proportion of respondents with cancer in each FRS ranged from 0-7%, with Nottinghamshire and Cumbria containing the largest proportion of respondents with cancer (note - as a % of total survey respondents from those areas, not as a % of total FRS headcount).

 **Figure S9: Geographic Distribution of Cancer Diagnoses in the UK Fire and Rescue Service.**The proportion of total survey respondents from each FRS who were diagnosed with cancer after joining the Service.

**Personal Contamination**

**
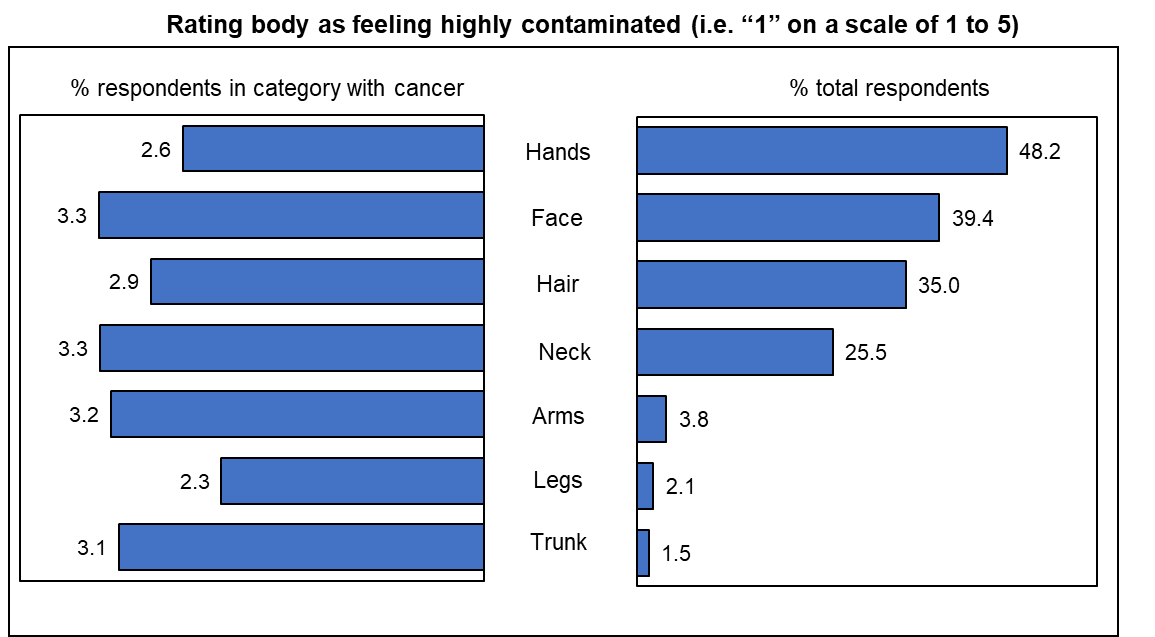
**

**Figure S10: Firefighters’ ratings of various body parts as feeling “highly contaminated”.**(Left) proportion of firefighters who rated each body part as feeling highly contaminated who were diagnosed with cancer after joining the Fire and Rescue Service. (Right) proportion of *total* surveyed firefighters who rated each body part as feeling highly contaminated (i.e. 1 on a scale of 1 to 5).
